# Changes of EEG band oscillations to tonic cold pain and the behavioral inhibition and fight-flight-freeze systems

**DOI:** 10.1017/pen.2019.9

**Published:** 2019-11-26

**Authors:** Vilfredo De Pascalis, Paolo Scacchia, Beatrice Papi, Philip J. Corr

**Affiliations:** 1 Department of Psychology, La Sapienza University of Rome, Rome, Italy; 2 Department of Psychology, City, University of London, London, UK

**Keywords:** tonic cold-pain, electroencephalography (EEG), behavioral inhibition system, fight-flight-freeze system

## Abstract

Using electroencephalography (EEG) power measures within conventional delta, theta, alpha, beta, and gamma bands, the aims of the current study were to highlight cortical correlates of subjective perception of cold pain (CP) and the associations of these measures with behavioral inhibition system (BIS), fight-flight-freeze system (FFFS), and behavioral approach system personality traits. EEG was recorded in 55 healthy right-handed women under (i) a white noise interruption detection condition (Baseline); (ii) enduring CP induced by the cold cup test. CP and Baseline EEG band power scores within conventional frequency bands served for covariance analyses. We found that: (1) higher Pain scorers had higher EEG beta power changes at left frontal, midline central, posterior temporal leads; (2) higher BIS was associated with greater EEG delta activity changes at parietal scalp regions; (3) higher FFFS was associated with higher EEG delta activity changes at temporal and left-parietal regions, and with lower EEG gamma activity changes at right parietal regions. High FFFS, compared to Low FFFS scorers, also showed a lower gamma power across the midline, posterior temporal, and parietal regions. Results suggest a functional role of higher EEG beta activity in the subjective perception of tonic pain. EEG delta activity underpins conflict resolution system responsible for passive avoidance control of pain, while higher EEG delta and lower EEG gamma activity changes, taken together, underpin active avoidance system responsible for pain escape behavior.

Experimental tonic pain in a non-clinical sample is a model that resembles clinical chronic pain and serves as a useful tool to examine underpinning brain mechanisms (Huber, Bartling, Pachur, Woikowsky-Biedau, & Lautenbacher, 2006; Nir, Sinai, Moont, Harari, & Yarnitsky, 2012). A plethora of electroencephalography (EEG) studies reported decreases of EEG alpha oscillations at around 10 Hz to tonic pain (Chang, Arendt-Nielsen, & Chen, 2002a, 2002b; Chen & Rappelsberger, 1994; Dowman, Rissacher, & Schuckers, 2008; Nir et al., 2012; Peng, Hu, Zhang, & Hu, 2014; Shao, Shen, Yu, Wilder-Smith, & Li, 2012), whereas other investigators obtained increases in the magnitude of gamma oscillations (30–100 Hz) (Dowman et al., 2008; Peng et al., 2014; Schulz et al., 2015; Veerasarn & Stohler, 1992). However, little research effort has been devoted to study how individual differences in personality traits modulate EEG oscillations during tonic pain experience.

Among the neurophysiological-based personality theories that could potentially play an important role in pain experience is the revised reinforcement sensitivity theory (rRST) of personality (Corr, 2008; Corr & McNaughton, 2012; Gray & McNaughton, 2000; McNaughton & Corr, 2008) that represents a reconceptualization of the RST originally formulated by Gray (1982, 1990). The behavioral inhibition system (BIS) and behavioral approach system (BAS) represent two neurophysiological brain systems that regulate how individuals respond to signals of potential punishment and/or reward. Specifically, the BIS is seen to be activated by signals of punishment and non-reward, novel stimuli, or unconditioned/conditioned fear stimuli. Its activation facilitates behavioral orienting through novel stimuli with interruption and inhibition of the ongoing behavior. The BAS is an appetitive–motivational system that is thought to respond to signals of reward and non-punishment. The BAS is activated by reward consumption and conditioned signals of reward or non-punishment and the associated approach behavior and positive emotions. In the rRST, the BAS is reconceptualized as a multidimensional system (Corr, 2016). The two systems work independently, although they can interact. While Gray’s original conception of RST mentioned a less defined fight-flight system (FFS), rRST developed this system further into a fight-flight-freeze system (FFFS). The original conception of BIS dealt with responses to aversive stimuli, but in the rRST, the FFFS was primarily responsible for this role, while the BIS serves primarily to detect and resolve the conflict between BAS and FFFS. The FFFS encompasses functional behavioral responses to threat, including fighting the threat, fleeing in active avoidance, or freezing to avoid attracting the attention of the predator. Pain and other aversive treat stimuli can require either avoidance or cautious approach. Simple avoidance is thought to be controlled by the FFFS. A cautious approach, induced by approach-avoidance conflict, is thought to initiate a conflict resolution process in the BIS. This resolution process could increase behavioral inhibition, negative bias, arousal, attention, and risk assessment (McNaughton & Corr, 2004).

Recent neuroimaging research has highlighted that BAS, but not BIS sensitivity of the Carver and White BIS/BAS scale (Carver & White, 1994), is positively associated with μ-opioid receptor availability in frontal cortex, cingulate cortex, insula amygdala, ventral, striatum, and brainstem, indicating that endogenous opioid system underlies BAS (Karjalainen et al., 2016). Although abovementioned studies indicate that pain responses may represent a form of responding to negative stimuli, the underlying brain mechanisms of the immediate effects of pain and their relation with motivational personality traits have been poorly understood. This is mainly due to the fact that, in motivational and physiological pain research, Carver and White BIS/BAS scale (Carver & White, 1994) is the most extensively used personality questionnaire, which is limited to only two measures of BIS and BAS. In addition, the BAS scale has no clear theoretical justification for its subdivision in three components, that is, drive, reward responsiveness, and fun seeking (for review, see Harmon-Jones, Harmon-Jones, & Price, 2013). However, the major problem with this questionnaire is the lack of separation of FFFS and BIS (Corr, 2016), which may account for inconsistent findings when relating BIS scale to placebo and nocebo effects (Corsi & Colloca, 2017). For example, Peciña et al. (2013) found that neuroticism was a negative predictor, while ego-resilience and agreeableness were positive predictors of pain reduction magnitude. Coen et al. (2011) found no influence of neuroticism on pain perception, whereas they found a positive correlation between brain activity and neuroticism during pain anticipation in regions associated with emotional and cognitive pain processing, including the parahippocampus, insula, thalamus, and anterior cingulate cortex (ACC). In contrast, these regions showed a negative correlation with neuroticism during pain perception. Further, neuroticism was also negatively correlated to ventral ACC activity when shocks were expected (Kumari, Das, Wilson, Goswami, & Sharma, 2007). Overall, BIS and FFFS-related personality traits were found positively correlated with ACC and posterior cingulate cortex (PCC) reactivity in response to negative events, and negatively with PCC activity while anticipating a negative event (for review, see Kennis, Rademaker, & Geuze, 2013).

Consistent with theoretical and empirical considerations of the rRST, a new questionnaire has been proposed, namely the Reinforcement Sensitivity Theory of Personality Questionnaire (RST-PQ; Corr & Cooper, 2016), developed on the basis of qualitative responses to defensive and approach scenarios. The RST-PQ highlighted a robust six-factor structure: two unitary defensive factors, the FFFS related to fear and the BIS related to anxiety, and four BAS facets (Reward Interest, RI; Goal-Drive Persistence, GDP, Reward Reactivity, RR; Impulsivity, Imp). The RST-PQ allows the separation of GDP, RI, and RR from Imp sub-factors of the BAS, making possible to test the unique predictive power of each sub-factor. Reward theory distinguishes between the anticipation of reward, closely linked to the motivation to obtain the reward, and the actual hedonic experience of reward (“wanting” vs. “liking”) (Berridge, 1996; Berridge, Robinson, & Aldridge, 2009). Whereas the “liking” component is associated with striatal opioids (Peciña & Berridge, 2000), “wanting” seems to be related to dopaminergic neurotransmission in the ventral striatum (Wyvell & Berridge, 2000). Individuals differ with respect to their sensitivity to reward and reward-predicting cues (Beaver et al., 2006; Cohen, Young, Baek, Kessler, & Ranganath, 2005; Shoaib, Spanagel, Stohr, & Shippenberg, 1995) and, in particular, “wanting” personality traits as assertiveness and reward sensitivity (i.e., drive and interest to achieve a reward) are associated with dopaminergic neurotransmission (DeYoung, 2010; Yacubian et al., 2007). The magnitude of opioid as well as dopamine release in the ventral striatum is related to the amount of pain relief (Scott et al., 2007, 2008; Zubieta et al., 2005), while change in the actual enjoyment of reward once it is achieved (“liking”) seems to be more closely related to opioidergic than to dopaminergic neurotransmission (Drago, Caccamo, Continella, & Scapagnini, 1984; Schweinhardt, Seminowicz, Jaeger, Duncan, & Bushnell, 2009; Shimizu et al., 2004). In this vein, since RI and GDP components of the BAS are conceptualized to serve the early stages of approach behavior (wanting or reward anticipation), these traits can be seen as the approach components linked to the dopaminergic activity. Additionally, as RR and Imp facets of the BAS are thought to serve the emotional excitement to reward, these traits are likely to depend from the function of the endogenous opioid system, which is mainly activated by the final biological reinforcer (Karjalainen et al., 2016; Peciña et al., 2013).

In the RST-PQ, the BIS and FFFS measures are postulated to have different functional properties and distinct neuropsychopharmacological bases (Corr & McNaughton, 2012; McNaughton & Corr, 2008) and separate sources of aversion (Perkins, Kemp, & Corr, 2007). However, we do not currently have specific biological markers that can be used to distinguish these sources in humans. According to McNaughton and Corr (2008), these two dimensions account for the differentiation between different defensive behaviors and involve somewhat different neural networks, especially with active versus passive avoidance. Serotonergic and noradrenergic fibers that essentially mediate global threat sensitivity are seen to modulate all the structures controlling defense (for more details, see Corr, DeYoung, & McNaughton, 2013).

EEG research on RST-related personality primarily attempted to link lateral frontal cortex, especially on the right, with avoidance-related processing mainly using Carver and White (1994) BIS/BAS scales to resting EEG alpha activity but inconsistent findings were found (for review, see Kennis, Rademaker, & Geuze, 2013; Wacker, Chavanon, & Stemmler, 2010). Original studies which related the BIS subscale with right frontal activity did not consider that Carver and White (1994) BIS scale was developed with only one general avoidance system in mind. However, research has linked right frontal activity to BIS-related states of response inhibition, and regulatory control. Enhanced activity in the right inferior frontal gyrus, following transcranial direct current stimulation, did produce greater response inhibition in a stop-signal task (Jacobson, Javitt, & Lavidor, 2011; Stramaccia et al., 2015) and, conversely, lesions of the right prefrontal cortex led to poor inhibition in a stop-signal task (Aron, Fletcher, Bullmore, Sahakian, & Robbins, 2003). Kelley and Schmeichel (2016) found the right frontal cortex involved in the inhibition of both approach and avoidance behavior, a key function of the BIS. Research using large samples of resting data has demonstrated greater BIS-anxiety related to the greater relative right frontal activity (De Pascalis, Fracasso, & Corr, 2017; Neal & Gable, 2016, 2017). Knyazev et al., using resting EEG data, also found that the relative prevalence of parietal alpha power and reduction in delta power were associated with higher BIS/N individuals, whereas relative prevalence of delta oscillations, mostly in the frontal region, predicted higher BAS impulsive individual (Knyazev, 2006; Knyazev & Slobodskaya, 2003). Other authors reported a negative association between theta power and neuroticism (Chi et al., 2005). A large literature suggests that enhanced theta activity in response to emotional stimuli is an index of perceived motivational salience, that is, the significance of a stimulus to the individual (Güntekin & Başar, 2014; Knyazev, 2007; Knyazev, Slobodskoj-Plusnin, & Bocharov, 2009). Frontal theta is generated in the ACC, which is crucial in the evaluation of stimulus salience in order to drive behavior (Bush, Luu, & Posner, 2000; Pizzagalli, Oakes, & Davidson, 2003). Andersen, Moore, Venables and Corr (2009) found theta band power especially responsive to anxious ruminative thinking and consistent with the model of recursive processing between the hippocampus and neocortex during goal-conflict resolution proposed by Gray and McNaughton (2000). Higher theta power reactivity to response execution during goal conflict in higher BIS participants was later reported by Moore, Mills, Marshman and Corr (2012) in a continuous monitoring task. Right frontal theta power has been found to increase in both conflict- and loss-induced theta power and associated with higher neuroticism scores (Neo & McNaughton, 2011). Very recently, we obtained heart-rate variability and conventional EEG band power measures during cold pain (CP) and placebo analgesia to identify RST-PQ traits that predict placebo analgesic responding. We found that a linear compound of HR slowing and higher EEG delta activity during placebo analgesia explained a substantial proportion of the variance in placebo analgesic responses, wherein RI had a significant mediating effect. These findings parallel our previous observations of reduced current density in the primary somatosensory cortex in higher total BAS and RI participants (De Pascalis & Scacchia, 2017a, 2017b). Other studies have outlined delta oscillations as a correlate of basic homeostatic and motivational processes as those involved in the detection of motivationally salient stimuli of reward and defensive mechanisms associated with pain and anxiety (Knyazev, 2007, 2012; Knyazev et al., 2009) and behavioral inhibition (Harmony, 2013; Kamarajan et al., 2004; Knyazev, 2007; Putman, 2011).

To date, research using primarily Carver and White (1994) BIS scale has not identified a neurocognitive correlate of this trait in humans (Kennis et al., 2013; Wacker et al., 2010). Thus, on the basis of the abovementioned EEG-pain findings, the aims of the present study were to detect, among EEG delta, theta, alpha, beta, and gamma bands, the brain rhythm sensitive to tonic CP and to highlight those sensitive to individual differences in pain and distress sensations. A further aim was to evaluate the link of both BIS and FFFS, as measured by the RST-PQ, with pain and distress sensations and to identify EEG band regional rhythms that can differentiate the defensive systems in terms of the BIS and FFFS. In line with Gray and McNaughton (2000) view, during pain, we expected a positive link between EEG theta activity and BIS scores as well as between delta activity and BIS scores. Finally, assuming that higher FFFS scorers should be prone to avoid painful stimulation, or to pay less attention to painful stimulation, we expected a reduced activity within the high-frequency EEG bands in these individuals. Finally, considering that BAS trait and its facets are activated by reward, we did not expect a link between pain sensation or EEG activity and these personality measures.

## 1. Methods

### 1.1 Participants

A total of 60 right-handed women (*M* = 23.8, *SD* = 2.1 years, range 19–32 years) student volunteers participated in the study, 4 of them were excluded for large EEG artifacts and 1 for presenting outliers, leaving 55 participants available for data analyses. We tested our hypotheses only in women since a body of literature clearly suggests that men and women differ in their responses to pain, with women being more sensitive (Bartley & Fillingim, 2013; Berkley, 1997). Further, to avoid individual differences in pain sensitivity due to menstrual pain, participants who were in a menstrual period were invited for the EEG recordings between the 5th and 11th day after the onset of menses. The study was approved by the institutional review board of the Department of Psychology according to Helsinki Declaration. Participants signed approved informed consent forms.

### 1.2 Questionnaires

The handedness of participant was assessed using the Italian version of the Edinburgh Inventory Questionnaire (Oldfield, 1971). Participants also completed the RST-PQ (Corr & Cooper, 2016). This tool measures three major systems: *FFFS* (related to fear); *BIS* (related to neuroticism/anxiety); *BAS*. The BAS, namely the total BAS, is a composite of four subscales Reward Reactivity (BAS-RR), Impulsivity (BAS-IMP), Goal-Drive Persistence (BAS-GDP), and BAS-Reward Interest (BAS-RI). Just before starting the EEG recordings, participants completed the State Anxiety Inventory (Spielberger, Gorsuch, Lushene, Vagg, & Jacobs, 1988) to measure state anxiety.

### 1.3 Experimental procedure

**Figure 1.**
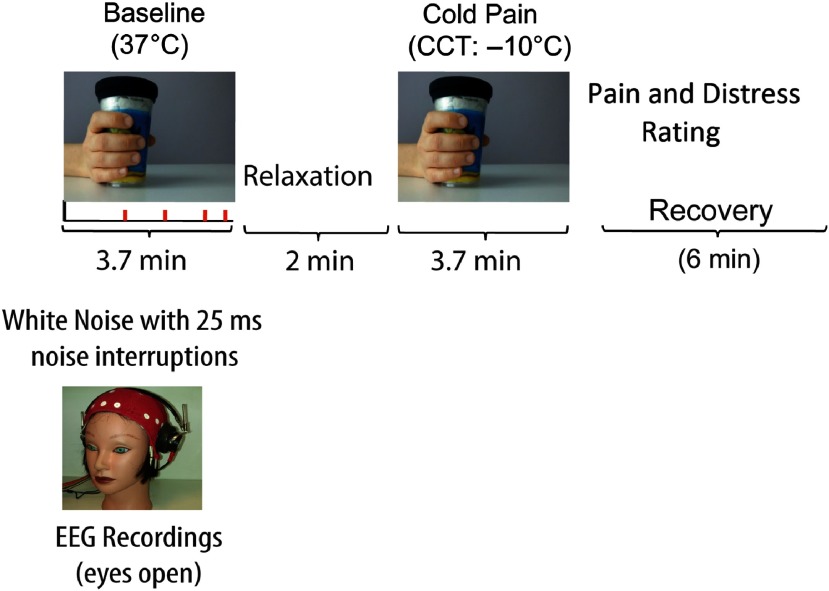
Schematic diagram depicting experimental treatments and procedure. Left quadrant in the panel shows a Baseline condition during which participants had to detect interruptions of a continuous white noise (Baseline). Right quadrant in the panel shows enduring CP induced by the CCT. Treatments were administered in counterbalanced order across participants. Following CP treatment, participants rated the intensity of experienced pain and distress sensation.

Participants came to the laboratory and consented to participate. They first completed the handedness and personality questionnaires and, then, an electro cap for EEG recordings was fitted. Participants completed an averaged time of about 13 min of EEG recordings during two treatments with eyes open (see Figure 1).

CP was induced by administration of the cold cup test (CCT; Chen, Chang, & Arendt-Nielsen, 2000; De Pascalis & Scacchia, 2019). The CCT can be considered as a variation of the ice-water cold-pressor test. This CCT proved to be convenient and consistent across the testing conditions. Participants received two treatments of 3.7 min each: (i) no-pain active Baseline, requiring to hold, in the right hand, a cup at about 37°C while listening, via binaural headphone, a continuous broadband white noise (70 dB, 0–44 kHz) wherein there were eight short interruptions (25 ms, rise time = 5 ms), randomly distributed within the Baseline time; and (ii) CCT, requiring a natural holding in the right hand of a tin-plastic chilled cold cup at −10°C. During the Baseline condition, the participant was required to pay attention to the ongoing white noise and to count the number of any possible changes in sound interruption, if any. As soon as the Baseline period was over, participants had to report verbally the number of white noise interruptions they detected. This Baseline condition was attempted to minimize the variability in arousal, attention, and vigilance both between and within participants by the auditory task. According to previous research (Dowman et al., 2008; Shao et al., 2012), the active non-pain condition should represent a better Baseline condition in contrast to the CP condition than the classic passive no-task control condition. Just at the end of CP treatment, the participant was required to rate the perceived pain and distress scores (NPS and NDS) on two separate 11-point (0–100) numerical rating scales (NRSs) (Jensen, Karoly, & Braver, 1986). The NRS for the perceived pain intensity was as follows: 0 = no pain, 10 = barely noticeable pain, 50 = mild pain, and 100 = maximum pain tolerable. The NRS for the perceived pain distress was as follows: 0 = neutral, 1 = barely distressing, 5 = distressing, and 10 = worst distressing imaginable. CP and Baseline treatments were administered in counterbalanced order across participants. In the cases in which Baseline preceded CP treatment, a 2-min relaxation period was given between treatments, in the opposite cases, the relaxation period was of 6 min.

### 1.4 EEG recording and processing

Participants were seated in a semi-reclined position inside a quiet dimly lit room. They were fitted with a pure-tin electrode electro-cap (Electro-Caps, Eaton, OH, USA) using an electrode placement based on the 10–20 system with a ground electrode mounted between FPz and Fz. Scalp EEG was recorded from 30 scalp sites (Fp1, Fp2, F7, F8, F3, F4, FT7, FT8, T3, T4, FC3, FC4, C3, C4, CP3, CP4, TP7, TP8, T5, T6, P3, P4, O1, O2, Fz, FCz, Cz, CPz, Pz, Oz) and referenced online to digitally linked ears [(A1 + A2)/2].

Bipolar horizontal and vertical electrooculograms (EOG) were recorded respectively from the epicanthus of each right and left eye, and from supra- and infra-orbital positions of the right eye using standard tin electrodes. Electrode impedances were kept under 5 kΩ, with homologous sites kept within 1 kΩ of one another. Data were collected using a 40-channels Neuroscan NuAmp amplifier unit (El Paso, TX, USA) settled in DC mode with a gain of 200 (100 for eye channels) and a band-pass of 0.01–75 Hz (Butterworth zero phase filter with 24 dB/octave roll off), notch filtered at 50 Hz (range 5 Hz), and digitized at 1000 Hz. EEG time series were then re-referenced to a common average reference and segmented into consecutive 2-s intervals. In order to eliminate artifacts, all data were offline visually inspected and hand-corrected for eyeblink artifacts using Brain Vision Analyzer 2.1 software. Eye-movement artifacts were removed by extracting 1–3 out of 30 independent components (IC; using Infomax algorithm, Brain Products; Vision Analyzer 2.01, Gilching, Germany) that clearly represented vertical and horizontal eye movements and had been identified by visual (topographic) inspection of the independent component analysis (ICA) maps and comparisons with the EEG and EOG time series (Delorme, Sejnowski, & Makeig, 2007; Olbrich, Jödicke, Sander, Himmerich, & Hegerl, 2011). Due to the influence of ICA correction on coherence measures (Castellanos & Makarov, 2006; Olbrich et al., 2011), only ICs without visible neural activity were discarded. Any segment that still contained muscle, movement, sweating, or eye-movement artifacts, as revealed by a visual inspection by two experienced clinical raters, was excluded from further analysis (no subject had more than 15% artifacts). A frequency resolution of 0.5-Hz steps for assessment of the different EEG bands was obtained. These 2-s EEG epochs were exported for further analysis.

### 1.5 EEG quantification

The data were parsed into 2-s epochs through a Hamming window, which was specified to diminish the signal on 10% at the borders of the epoch, to prevent spurious estimates of spectral power. After a visual re-examination for muscle, eye, movement, sweat, and technical artifacts, artifact-free epoch data were identified and, to facilitate later processing, downsampled to 256 Hz. An average of 90.7 (*SD* = 5.8) and 88.2 (*SD* = 7.1) of usable non-overlapping epochs were obtained, respectively, for Baseline and Pain conditions in each participant. Fast Fourier transform algorithm was used to perform EEG frequency analysis, with 2-s interval on the EEG signal, in all scalp locations. The bands inspected were the traditional delta (0.5–3.75 Hz), theta (4–7.75 Hz), alpha (8–12.75 Hz), beta (13–35.75 Hz), and gamma (36–45 Hz), and power values were averaged across epochs. Since the CP has a strong negative valence, which is known to increase over the course of time (Streff, Kuehl, Michaux, & Anton, 2010), power spectra were computed 30 s after that painful stimulation had started. The same starting time of 30 s was used for EEG analysis during the Baseline. Power values were natural-logarithm (ln) transformed to normalize the data (Gasser, Bächer, & Möcks, 1982). These values were then used to calculate EEG band power changes from Baseline by subtracting EEG band values during Baseline from those obtained during Pain. Based on previous pain study reports (Apkarian, Bushnell, Treede, & Zubieta, 2005; Dowman et al., 2008; Koessler et al., 2009; Okamoto et al., 2004), we selected for statistical analyses the following 15 scalp recording sites: F7, F3, Fz, F4, F8, T3, C3, Cz, C4, T4, T5, P3, Pz, P4, and T6.

### 1.6 Statistical analyses

To evaluate the relation of pain with personality measures of interest, we first calculated the zero-order correlation of NPS, NDS, state anxiety, and RST-PQ traits. Significance of these correlations was assessed using the bias-corrected bootstrap method, which is effective in controlling for type 1 errors associated with multiple comparisons (Efron & Efron, 1982; Efron & Gong, 1983). For each correlation, we also computed the 95% confidence intervals (CI) using bootstrap resampling (5000 samples, bias-corrected confidence limits). All significant coefficients with an associated CI that did not include zero were considered statistically significant (*P* < .05).

To evaluate the effect of pain and personality on EEG activity, separate ANCOVAs were applied (SAS-9.4 system) using each of the associated pain and personality measures as a covariate. Condition (Baseline, CP) and Topography served as within subject’s factors. Within Topography, sagittal plane (anterior-frontal [F7, F3, Fz, F4, F8], temporo-central [T3, C3, Cz, C4, T4] and temporo-parietal [T5, P3, Pz, P4, T6] regions) and coronal plane (left-1 [F7, T3, T5], left-2 [F3, C3, P3], midline [Fz, Cz, Pz], right-1 [F4, C4, P4], right-2 [F8, T4, T6] regions) were repeated-measures factors. An *α* level of .05 was used for all analyses. Huynh-Feldt adjustments were used when the assumption of sphericity was violated. To control for false-positive errors, significance levels were corrected using the false discovery rate (FDR) method (“proc multitest,” SAS-9.4; Benjamini & Hochberg, 1995). Only for graphical illustrations, and to understand the direction of changes of significant main and/or interaction effects involving NPS or personality traits of interest, we applied separate median splits on these self-report measures. Participants were considered as belonging to either group “high” (Hi) or “low” (Lo) when their scores on the pain and personality measures were above or below the median. Pain and personality scores falling on the median were excluded.

## 2. Results

### 2.1 Correlations among RST-PQ personality traits and NPS and NDS measures

Pearson correlation coefficients (bias-corrected bootstrap method) among RST-PQ personality traits, state-anxiety, NPS, and NDS ratings together with descriptive statistics are reported in Table 1. Correlations among personality measures confirm the pattern of originally reported associations (Corr & Cooper, 2016), while no significant correlations were found between RST-PQ traits as well as state anxiety and pain sensation measures.

**Table 1.** Pearson correlation coefficients and descriptive statistics for rRST personality traits and numerical pain and distress score (NPS and NDS). Bootstrapped 95% CI is reported in parentheses (*N* = 55)

Variable	1	2	3	4	5	6	7	8	9
1. BIS	1								
2. FFFS	0.47*** (0.36, 0.59)	1							
3. BAS-RR	−0.16 (−0.37, 0.01)	0.11 (−0.19, 0.13)	1						
4. BAS-IMP	−0.13 (−0.21, 0.12)	−0.17 (−0.35, 0.08)	0.46*** (0.30, 0.65)	1					
5. BAS-GDP	0.11 (−0.03, 0.24)	−0.01 (−0.17, 0.23)	0.37** (0.31, 0.52)	0.12 (−0.06, 0.13)	1				
6. BAS-RI	−0.37** (−0.53, −0.19)	−0.14 (−0.19, 0.21)	0.47** (0.35, 0.57)	**0.51**† (0.35, 0.68)	**0.52**† (0.32, 0.65)	1			
7. NPS	0.00 (−0.13, 0.18)	−0.00 (−0.15, 0.22)	0.09 (−0.03, 0.21)	0.24 (−0.00, 0.28)	0.05 (−0.09, 0.28)	0.06 (−0.12, 0.21)	1		
8. NDS	0.07 (0.15, 0.26)	0.06 (−0.06, 0.25)	0.21 (0.09, 0.31)	0.24 (0.12, 0.35)	0.06 (−0.07, 0.26)	0.17 (−0.14, 0.23)	**0.66**^†^ (0.55, 0.80)	1	
9. STAI-Y1	0.42** (0.35, 0.57)	0.24 (0.00, 0.26)	−0.33* (−0.43, −0.20)	−0.31* (−0.39, −0.18)	−0.20 (−0.26, −0.01)	−0.28* (−0.35, −0.11)	−0.10 (−0.12, 0.15)	−0.01 (−0.11, 0.21)	1
Mean	52.1	26.5	29.8	17.5	23.1	19.7	55.4	36.8	34.9
*SD*	9.3	5.3	3.8	3.9	3.9	3.5	21.9	26.5	5.9
Range	32–79	17–40	24–39	10–29	10–30	11–28	17–93	0–94	21–47
Cronbach’s *α*	.89	.81	.79	.76	.83	.78	–	–	.82

BIS, behavioral inhibition system; FFFS, fight-flight-freeze system; T-BAS, total score for behavioral approach system; GDP, goal-drive persistence; RI: reward reactivity; Imp: impulsivity.

*Notes:* Personality Measures: Reinforcement Sensitivity Theory Personality Questionnaire (RST-PQ; Corr & Cooper, 2016).

NPS and NDS: 0–100 Numeric Rating Scale (Jensen, Karoly, & Braver, 1986). STAI-Y1: State Anxiety (Spielberger et al., 1988).

*P < .05; **P < .01; ***P < .001; ^†^*P* < .0001.

Bold entries indicate significant values.

### 2.2 Pain perception, BIS, FFFS, and relevant EEG band power measures

**Figure 2.**
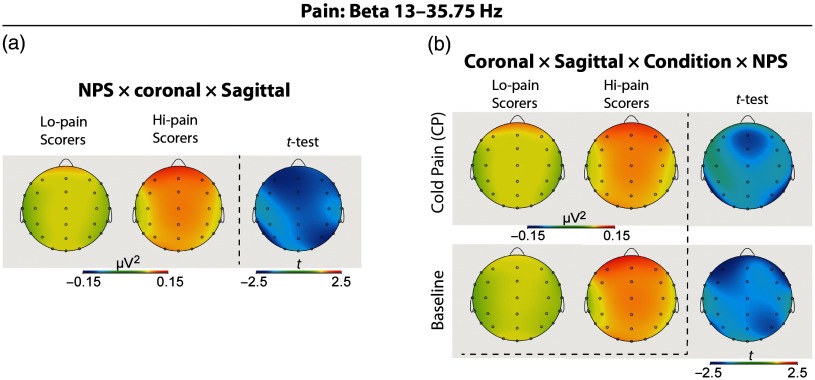
Topographic patterns of significant ANCOVA effects (individual pain score (NPS) as a covariate) on beta power for (a) High-Pain vs. Low-Pain scorers; (b) Interaction of NPS with Topography and Condition (Baseline, CP). Independent *t*-test topographies compared High-Pain vs. Low-Pain scorers.

**Figure 3.**
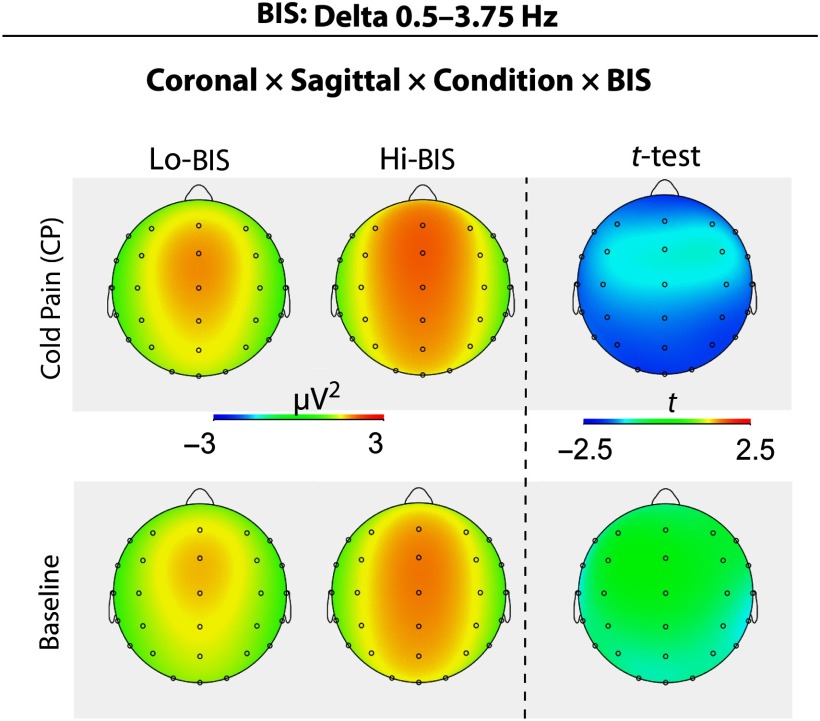
Delta power topographic patterns of a significant ANCOVA interaction of BIS trait (covariate) with Topography (Sagittal, Coronal plane) and Condition (Baseline, CP). Independent *t*-test topographies compared Low BIS vs. High BIS scorers.

**Figure 4.**
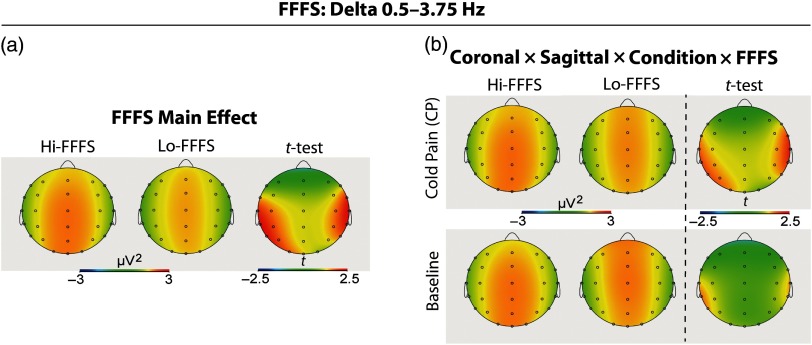
Topographic patterns of significant ANCOVA effects on delta power for (a) High FFFS vs. Low FFFS scorers; (b) Interaction of FFFS with Topography and Condition (Baseline, CP). Independent *t*-test topographies compared High FFFS vs. Low FFFS scorers.

**Figure 5.**
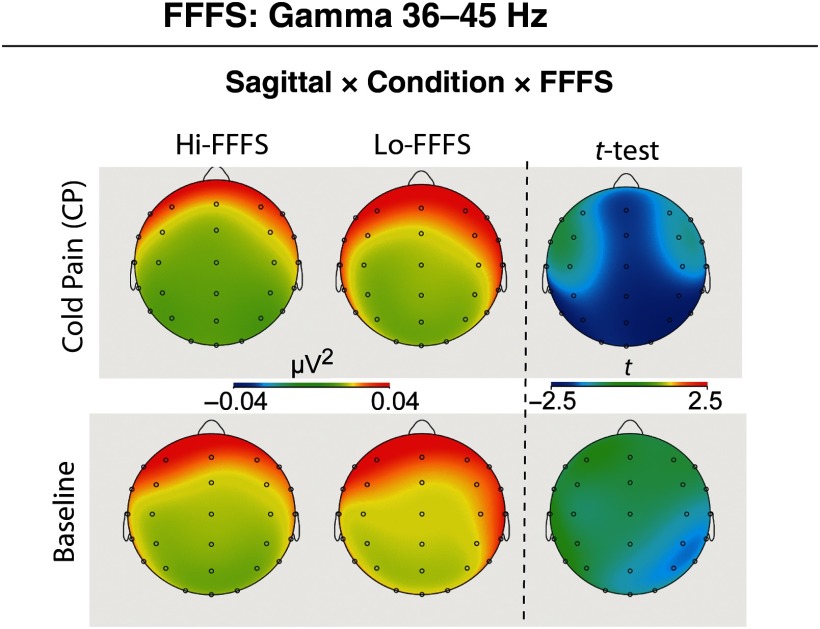
Gamma power topographic patterns of a significant ANCOVA interaction of FFFS trait (covariate) with Topography (Sagittal, Coronal plane) and Condition (Baseline, CP). Independent *t*-test topographies compared High FFFS vs. Low FFFS scorers.

For each of the EEG delta, theta, alpha, beta, and gamma band power measures, separate ANCOVAs were performed by separately using each of the self-reported measures of interest as a covariate (i.e., pain and distress ratings and RST-PQ traits). Results of the ANCOVAs are given in Table 2. Additionally, for graphical illustrations, and to display the direction of significant changes, *post-hoc t*-tests were performed based on a median split of NPS, BIS, and FFFS scores (see Figures 2–5). The number of individuals falling on the median was: 7 for NPS (*N* = 26 Hi-NPSs and *N* = 22 Lo-NPSs); 5 for BIS (27 Hi-BIS, 23 Lo-BIS); and 6 for FFFS (24 Hi-FFFS, 25 Lo-FFFS).

**Table 2.** *F*-, *P*- and 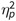-values for the main and interaction effects in the analyses of covariance with the factor Condition (Pain vs. Baseline), Coronal and Sagittal topography in the 2 × 3 × 5 factorial design for EEG band power measures

ANCOVA	Beta: covariate = NPS			Delta: covariate = FFFS			Delta: covariate = BIS			Gamma: covariate = FFFS		
2 Condition × 3 Sagittal × 5 Coronal × Covariate	*F*	*P*	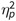	*F*	*P*	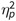	*F*	*P*	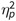	*F*	*P*	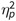
Covariate	4.05	.080	0.684	0.13	.850	0.002	**6.15**	**.048**	0.110	1.46	.5379	0.026
Condition	1.99	.194	0.031	1.48	.521	0.020	0.17	.811	0.003	1.08	538	0.019
Condition × Covariate	2.12	.194	0.031	1.00	.521	0.020	0.02	.881	0.000	1.11	.538	0.021
Coronal plane	0.38	.699	0.007	**19.75**	**.0001**	0.390	**33.70**	**.0001**	0.890	3.28	.1343	0.058
Coronal plane × Covariate	1.81	.194	0.021	1.23	.521	0.023	3.79	.059	0.071	2.23	.2633	0.029
Coronal Plane × Condition × Covariate	1.19	.343	0.022	1.23	.521	0.023	2.87	.093	0.051	**10.40**	**.0001**	0.164
Sagittal plane	8.45	.013	0.116	0.20	.850	0.004	0.70	.560	0.013	**7.02**	**.013**	0.117
Sagittal plane × Covariate	3.70	.078	0.080	0.01	.947	0.0002	0.12	.844	0.002	1.46	.549	0.021
Coronal × Sagittal	**4.81**	**.031**	0.075	1.97	.947	0.037	2.46	.093	0.046	0.75	.758	0.014
Sagittal × Condition × Covariate	**5.69**	**.030**	0.110	0.94	.521	0.018	2.33	.188	0.043	1.12	.538	0.019
Coronal × Sagittal × Condition	**4.73**	.0001	0.143	1.97	.394	0.037	1.91	.188	0.034	0.69	.7583	0.012
Coronal × Sagittal × Covariate	**2.69**	**.043**	0.034	1.30	.521	0.022	1.95	.176	0.037	0.50	.858	0.008
Coronal × Sagittal × Covariate × Condition	**3.04**	.031	0.094	**3.31**	.007	0.090	**2.44**	**.049**	0.041	0.74	.7583	0.014

*P* values are corrected using FDR method. Bold entries indicate significant values.

### 2.3 Pain perception and EEG beta band power

Using NPSs as a covariate, we obtained, for beta band power, a significant Coronal by Sagittal by NPS interaction, revealing a significantly higher beta power in Hi-Pain than Lo-Pain scorers at frontal and right-parietal leads (see Table 2 and Figure 2(a)). In addition, for beta power, the Coronal by Sagittal by NPS by Condition interaction was also significant. This interaction disclosed a significantly higher beta power in High-Pain scorers at Fz, FCz, Cz, F8, T5, and T6 leads during CP and at F3, F4, FC4, and P4 leads during Baseline (Table 2 and Figure 2(b)). No significant effects involving distress rating scores were observed for the EEG band power measures of interest.

### 2.4 Effects of BIS and FFFS on EEG delta and gamma powers

No significant effects involving any of RST-PQ traits were obtained for theta and alpha band power measures.

The ANCOVA performed on delta power scores, using BIS as a covariate, disclosed a significant effect for Coronal plane followed by a significant four-way interaction for Coronal by Sagittal by BIS by Condition. These effects revealed that during CP condition there was a higher delta power in Hi-BIS as compared to the Lo-BIS participants at P3, Pz, and P4 leads (Table 2 and Figure 3).

A similar analysis conducted on delta power scores, using FFFS trait as a covariate, yielded a main effect for this trait, indicating that Hi-FFFS participants had a higher delta power than Lo-FFFS ones (Table 2 and Figure 4(a)). Moreover, the two-way Coronal by FFFS was near the significance level (FDR corrected *P* = .059), and four-way Coronal by Sagittal by Condition by FFFS interactions were all significant (Table 2). These effects indicated that Hi-FFFS scorers, for the CP condition, had a relatively greater delta power than Lo-FFFS scorers at T3, T5, P3, T4, and T6 leads, while for the Baseline these differences disappeared (Figure 4(b)).

Finally, ANCOVA on EEG gamma power disclosed significant effects for both Sagittal and Coronal planes and a significant three-way Coronal by Condition by FFFS interaction (Table 2). This interaction indicated that, during the CP condition, Hi-FFFS participants had a lower gamma power than Lo-FFFS ones across all midline sites (Fz, Cz, Pz) and left and right posterior temporal (T5, T6) and parietal leads (P3, P4) (Figure 5).

## 3. Discussion

Findings from the present study did not disclose significant EEG band oscillation changes between CP and Baseline condition. This means that our results did not support findings from previous studies in which cold-pressor test led to an increase in delta (Chen, Dworkin, Haug, & Gehrig, 1989; Ferracuti, Seri, Mattia, & Cruccu, 1994; Huber et al., 2006), theta (Chen, Rappelsberger, & Filz, 1998; Russ, Campbell, Kakuma, Harrison, & Zanine, 1999), beta, and gamma activity (Chang et al., 2002b; Shao et al., 2012). These differences could be due to the fact that we used an active Baseline task during which participants had to focus attention on task-relevant stimuli (i.e., they had to detect and count changes in the ongoing white noise) which may have produced a general activation response in this experimental condition (Peng et al., 2014). However, in the current study, ANCOVA analyses on the conventional EEG band oscillation changes, induced by CP from Baseline, disclosed that (a) beta power change was sensitive to individual differences in pain perception, (b) delta power to individual differences in BIS levels, and both delta and gamma powers to individual differences in FFFS traits.

In terms of pain perception (NPS), the ANCOVA performed on EEG beta band power scores disclosed a main effect for the covariate NPSs, indicating higher frontal beta activity in High-Pain scorers compared to Low-Pain scorers. This analysis also showed that these individual differences were more pronounced at Fz, Cz, F8, T5, and T6 leads during CP condition and at F3, F4, and P4 leads during Baseline condition (Table 2 and Figure 2). In the whole, these findings suggest a functional role of EEG beta activity in representing subjective experiences of tonic pain. Specifically, our finding of enhanced beta activity in pain-sensitive individuals (Table 2) is in line with those reported in previous studies using long-lasting tonic pain stimulations wherein tonic pain was found associated with decreased alpha and increased beta activities (Chang et al., 2002a; Chang, Arendt-Nielsen, Graven-Nielsen, Svensson, & Chen, 2004; Chen & Rappelsberger, 1994; Giehl, Meyer-Brandis, Kunz, & Lautenbacher, 2014; Shao et al., 2012) and with higher beta power density findings (Huber et al., 2006; Ploner, Sorg, & Gross, 2017). Research has also demonstrated that beta and gamma band activities enhance with heightened attention to pain stimulus (Hauck, Lorenz, & Engel, 2007; Tiemann, Schulz, Gross, & Ploner, 2010), vary with conscious perception (Gross, Schnitzler, Timmermann, & Ploner, 2007) and attention effects of pain (Tiemann et al., 2010). Accordingly, our observations of enhanced relative fronto-temporal EEG beta activity to CP in Hi-Pain scorers may reflect the operation of an excitatory process employed for the encoding of subjective experiences of tonic pain. In contrast, the reduced beta activity in Lo-Pain scorers may reflect the disposition, in these individuals, toward an inhibitory attentional-shift from painful stimulus making a reduced pain perception. Moreover, the present data indicate that these EEG changes are specific to tonic CP, as compared to changes observed during a non-painful Baseline stimulation, this is since we did not find any significant association between NPSs and state anxiety or dispositional personality traits of interest.

In terms of individual differences in rRST traits, our statistical analyses disclosed that Hi-BIS, as compared to Lo-BIS participants, had a relatively higher EEG delta power increase during CP across frontal, temporal, and parietal leads (Table 2 and Figure 3). These relatively new findings appear in line with few reports suggesting delta responses as a modulator of signal detection and decision making (Başar, Başar-Eroglu, Karakaş, & Schürmann, 2001; Schürmann, Başar-Eroglu, Kolev, & Başar, 2001), with studies indicating a role of delta oscillations in the synchronization of brain activity with autonomic functions in higher emotional involvement such as pain and in anxiety disorders (Knyazev, 2012). Delta activity was also associated with the detection of motivationally salient stimuli of reward and ancestral defensive mechanisms (Knyazev, 2007; Knyazev et al., 2009) and to behavioral inhibition (Harmony, 2013; Kamarajan et al., 2004; Knyazev, 2007; Putman, 2011). More specifically, Kamarajan et al. (2004) reported suppressions of frontal delta and theta responses in alcoholic individuals which are likely to show deficits in cognitive functions that are mediated by these oscillatory processes. Thus, the higher relative delta power, we obtained during CP, in Hi-BIS participants, may reflect the enhanced adaptive attempt devoted by these individuals to resolve the conflict associated with tonic pain perception (Amodio, Master, Yee, & Taylor, 2008; De Pascalis, Varriale, & D’Antuono, 2010), vice versa, the relative lower delta power we did find in Lo-BIS participants may reflect the reduced tendency to process tonic CP stimulation in these individuals. However, this interpretation remains a purely speculative attempt to explain current results considering that we have not found any significant association between NPS and BIS, FFFS, or BAS traits. It should be noted that a lack of association between pain perception and anxiety-related traits is not new. For example, in a previous personality-pain study (Coen et al., 2011) no association between neuroticism and pain ratings has been reported during visceral pain perception, although a negative correlation between neuroticism and brain activity was obtained in regions associated with emotional and cognitive pain processing, including the parahippocampus, insula, thalamus, and ACC (Coen et al., 2011). Additionally, in a previous fMRI-pain study (Kumari, Das, Wilson, Goswami, & Sharma, 2007) neuroticism correlated positively with the ratings of fear of a shock and negatively with brain activity in the anterior and posterior cingulate, superior/middle temporal gyrus extending to the hippocampus, precuneus, putamen, thalamus, and middle occipital gyrus. These observations support the view of reduced processing of pain in subjects with higher levels of neuroticism, especially the anxiety component of this trait (Kumari et al., 2007). Further, fMRI findings (Bishop, Duncan, Brett, & Lawrence, 2004) found that higher state anxiety levels were associated with both less rostral ACC activity and reduced recruitment of lateral PFC, suggesting that higher state anxiety is associated with reduced top-down control over threat-related stimuli. Unfortunately, we failed to extend abovementioned findings to tonic CP stimulation since we did not find a significant association between state anxiety and EEG power changes within conventional frequency bands during CP. We also failed to find a significant association between midfrontal theta activity and BIS as reported in previous studies (Andersen et al., 2009; Cavanagh & Shackman, 2015; Gray & McNaughton, 2000; Moore et al., 2012; Neo & McNaughton, 2011). This may depend from differences in the type of task used between the current and previous studies. For example, while in previous studies the BIS–theta relationship was found for designed tasks requiring rapid resolution of a cognitive goal conflict (e.g., Moore, Gale, Morris, & Forrester, 2006; Moore et al., 2012; Neo & McNaughton, 2011), in the current study the conflict consisted in paying attention to a continuous white noise in order to rate it. Thus, further investigation is justified.

In terms of the FFFS trait, ANCOVA analysis disclosed that Hi-FFFS participants, compared to Lo-FFFS participants, had higher delta power changes to CP at right-temporal and left-parietal leads, whereas they had lower gamma power changes at midline fronto-central and right parietal leads (Table 2; Figures 4 and 5). It is important to note that in the present study painful stimulation cannot be avoided, and the main function of FFFS activation serves to actively avoid the treat (active avoidance; Corr & McNaughton, 2012). Considering that in previous studies delta activity was found associated with functional cortical deafferentation of sensory inputs that interfere with internal concentration necessary to accomplish a given task (Buzsáki, 2006; Harmony, 2013), our findings can be explained assuming that in higher FFFS individuals the active avoidance behavior is manifested through the increased delta power necessary to inhibit painful sensory afferences that interfere with concentration necessary to rate pain sensation. This finding is interesting and needs of further replications.

According to Botvinick (2007), pain falls into a class of conflicting signals, as monetary loss and negative feedback, which are registered within the ACC and weighted as an aversive or costly event. This would have a direct impact on decision making, influencing subsequent adaptive behavioral adjustments in avoidance-learning mechanisms. As previously stated, we believe that in Hi-FFFS participants were mainly involved spontaneous defensive avoidance mechanisms to facilitate their cognitive control of painful experience (Deakin & Graeff, 1991). In line with Botvinick (2007), Buzsáki (2006), Knyazev (2012), and Harmony (2013) suggestions, we think that in Hi-FFFS participants the relative increase in temporo-parietal delta activity taken together with the relative decrease in cortical gamma activity, across midfrontal, temporal, and parietal regions, can serve to activate active-avoidance control mechanisms in order to reduce focused attention on painful stimulation and to prevent negative outcome. These findings appear also in line with previous reports suggesting that a complex network, including sensory cortices, insula, hippocampus, amygdala, and periaqueductal gray, is activated to an incoming threat associated with painful stimulation (Corr & McNaughton, 2012; Faull & Pattinson, 2017; Mobbs et al., 2007).

It is important to underline that the present study has some limitations. First, the sample was restricted to right-handed women. Our findings thus may not be applicable to men or left-handed women. This is since it has been shown that right-handed females perceive a painful heat stimulus as more intense than do males (see, e.g., Paulson, Minoshima, Morrow, & Casey, 1998) and this is associated with greater activation in the contralateral thalamus and anterior insula. In addition, pain threshold and tolerance in response to submerging a hand in very cold-water baths have been found higher on the right hand in dextral subjects (Pud, Golan, & Pesta, 2009; Sarlani, Farooq, & Greenspan, 2003; Schiff & Gagliese, 1994). Second, this study was exploratory in nature since personality traits, as measured by the RST-PQ, have not yet been studied in relation to tonic CP and EEG oscillations. Third, participants rated subjective pain and distress intensity just at the end of the cold stimulation condition, and not continuously monitored during tonic CP stimulation. Thus, the results are tentative and need verification through additional research.
